# Effectiveness of Resistance Training on Fatigue in Patients Undergoing Cancer Treatment: A Meta-Analysis of Randomized Clinical Trials

**DOI:** 10.1155/2022/9032534

**Published:** 2022-08-08

**Authors:** Lily Berríos-Contreras, Rodrigo Cuevas-Cid, Luz Alejandra Lorca, Ivana Leão Ribeiro

**Affiliations:** ^1^Laboratorio de Investigación Clínica en Kinesiología, Departamento de Kinesiología, Facultad de Ciencias de la Salud, Universidad Católica del Maule, Talca, Chile; ^2^Hospital del Salvador, Servicio de Salud Metropolitano Oriente, Santiago, Chile

## Abstract

**Objective:**

To assess the effectiveness of the resistance training to improve fatigue levels in people with cancer who are enrolled in adjuvant and/or neoadjuvant treatment.

**Methods:**

MEDLINE, Web of Science, Embase, SPORTDiscus, LILACS, CENTRAL, and CINAHL databases were searched from May to December 7, 2021. Randomized clinical trials (RCT) that evaluate the effects of resistance training on fatigue levels in people undergoing cancer treatment were included. The PEDro scale was considered to assess methodological quality of studies, and the evidence was summarized through the GRADE system. The standardized average differences, effect size, and inverse variance model for meta-analysis were calculated.

**Results:**

Fifteen RCT for qualitative synthesis and thirteen for meta-analysis were selected. A moderate to high level of evidence of resistance training was identified to improve fatigue in people undergoing cancer treatment. Meta-analysis showed a significant reduction in fatigue (SMD = −0.31, CI 95% = −0.58, -0.12, *P* = 0.001) after 10 to 35 sessions of resistance training.

**Conclusion:**

The 10 to 35 sessions of resistance training are effective in reducing fatigue level in cancer patients who are undergoing cancer treatment and have a moderate level of quality evidence.

## 1. Introduction

According to the World Health Organization (WHO), cancer incidence and mortality increase each year, making it the second leading cause of death in the Americas after cardiovascular diseases. Cancer treatment can be neoadjuvant (presurgery) or adjuvant (postsurgery), which can consist of radiation therapy, chemotherapy, hormone therapy, androgen deprivation therapy, and hematopoietic call transplantation [1–3]. Cancer treatment is aimed at restoring the function of the organ affected by the malignant tumor [4] or controlling the progression of neoplasia through DNA damage and mitotic spindle inhibition of cancer cells [5, 6]. The adjuvant and/or neoadjuvant treatment increases cancer patients' survival; however, it can generate negative side effects such as muscle atrophy, loss of functionality, reduced mobility, and fatigue related to cancer [7].

Cancer patients can present a high degree of fatigue perception that can last 5 to 6 weeks after cancer treatment and drastically affect quality of life [8]. The mechanisms involved in fatigue have been associated with the release of proinflammatory cytokines by the malignant neoplasia and cancer treatment that alters the hypothalamic-pituitary-adrenal axis [9–11] and generates a dysfunction of the autonomic nervous system [12]. Fatigue is defined as a “sensation of tiredness or distressing, persistent, subjective exhaustion related to cancer or cancer treatment that is not proportional to recent activity and interferes with normal functioning” [13].

Resistance training in cancer patients improves quality of life and increases adherence and success of cancer treatment, in addition to contributing to the increase in lean mass and body weight, preventing muscle mass loss, reducing its side effects, and increasing survival [14–16]. There are two systematic reviews that reported the effects of exercise and physical activity on fatigue levels in cancer survivors and patients with metastasis [17, 18]; however, these studies did not include specific information regarding the dosage of resistance training and its effect during cancer treatment. The objective of this study is to evaluate the effectiveness of the resistance training (RT) to improve fatigue levels in people with cancer who are undergoing adjuvant and/or neoadjuvant treatment.

## 2. Material and Methods

### 2.1. Protocol and Registration

The present systematic review of randomized clinical trials and meta-analyses was performed according to the report of elements for systematic reviews and meta-analyses (PRISMA) [19] and Cochrane recommendations [20]. The reference number of the International Registry of Prospective Systematic Review (PROSPERO) is CRD 42020196180.

### 2.2. Search Strategy and Article Selection

Studies were selected if they met the following inclusion criteria based on publication types based on the participants, interventions, comparisons, outcomes, and study design (PICOS): (1) population: subjects over 18 years old with cancer who are undergoing cancer treatment; (2) intervention type: RT; (3) comparisons: other training type controls without intervention; (4) results: fatigue; and (5) studies: randomized clinical trials (RCT), published in English or Spanish. The articles were excluded if the sample consisted of people who were not undergoing cancer treatment.

An electronic search was performed until December 7, 2021, in the following electronic databases: MEDLINE (via PubMed), Embase, Web of Science, CINAHL, SPORTDiscus, LILACS, PEDro, Cochrane, and EBSCO. The following Medical Subject Headings (MeSH) terms were included and combined for sensitive searches: “Cancer”; “radiotherapy”; “chemotherapy”; “fatigue” and “resistance exercise”. The search strategy used in the MEDLINE was as follows: ((((fatigue) AND (cancer)) AND (radiotherapy)) OR (chemotherapy)) AND (resistance exercise). The strategy was adapted according to each database and is specified in a supplementary file (available here).

The articles were initially selected for screening of the title and the abstract, ending with a full text evaluation. Two evaluators (LB, RC) evaluated each scientific article independently. In case of disagreement or discrepancy in the inclusion or exclusion of any article, it was sent to an independent reviewer (IL), who decided in this respect to agree on its selection for the complete analysis of the article. The same process was performed to evaluate both methodological quality and level of evidence of included articles.

### 2.3. Data Extraction and Analyses

For data extraction, standardized forms adapted from the Cochrane Collaboration model [20] were used. The following aspects were considered to describe the characteristics of the studies: population (sex, age, number of participants per group, cancer type, and cancer treatment), intervention (type, duration, and frequency), and dosage of resistance exercise (type of external load, maximum repetition percentage (%1RM), increase load, number of sets, and repetitions). To describe the results of the selected studies, the following were considered: questionnaire type used to assess fatigue, effect size, and fatigue levels pre- and post-RT (variation percentage). The results of the perception of fatigue were evaluated according to total training time. Additionally, the effect size (ES) was calculated to determine the clinical relevance of the interventions with a 95% confidence interval, considering pre- and postintervention values. A small effect of the intervention was considered <20; a moderate effect, between 21 and 0.79; and a great effect, >0.80 [21]. The negative effect size represents a “worsening of symptoms” while the positive one represents an improvement [22]. All primary studies that presented descriptive values as mean and standard deviation were considered to ES calculation.

### 2.4. Quality Assessment

To evaluate the methodology quality and the risk of bias of the included RCT, we used the Physiotherapy Evidence Database (PEDro) scale [23]. The PEDro scale allows the bias rating of articles according to a cut-off score. The studies with a 10-9 score correspond to an excellent quality methodology, those with an 8-6 score correspond to a good methodology quality, those with a 4-5 score correspond to an acceptable methodology quality, and those that had a score lower than 4 correspond to a bad methodology quality [24, 25].

The Grades of Recommendation, Assessment, Development, and Evaluation (GRADE) scale was used to evaluate the level of evidence categorizing it into 4 levels: high, moderate, low, and very low [26]. GRADE considers 5 criteria: limitations in study design, indirectness of evidence, heterogeneity, imprecision of results, and publication bias. Each criterion is evaluated as follows: not serious, serious, and very serious considering a consensus between two raters [21]. The high characterization reflects that there is a high confidence in the coincidence between the real and the estimated effect; the moderate quality level corresponds to a moderate confidence in the estimate effect; that is, there is a possibility that the real effect is far from the estimated effect. On the contrary, a low and very low quality of evidence reflects that there is uncertainty regarding the intervention effects [26, 27].

### 2.5. Statistical Methods

A descriptive synthesis was performed considering the mean, standard deviation, distribution by frequency, and percentage of the results of the primary studies. In addition, the meta-analysis was calculated to determine the effects of resistance training on fatigue levels. The analysis was performed using means and standard deviations from each selected clinical trial. Results of some questionnaires indicated an increase in fatigue levels while others indicated a decrease, according to the direction of questions. The mean values of a group of studies were multiplied by -1 to ensure that all instruments point in the same direction [20]. The difference in standardized means and the 95% confidence interval (CI) were calculated using an inverse variance model of random effects for the meta-analysis, considering postintervention data. Heterogeneity data between studies was evaluated using the *I*^2^ statistic, and *P* value was set at 0.01. The statistical analysis was performed with five revisions of RevMan management software (version 5.3).

## 3. Results

### 3.1. Study Selection

**Figure 1:**
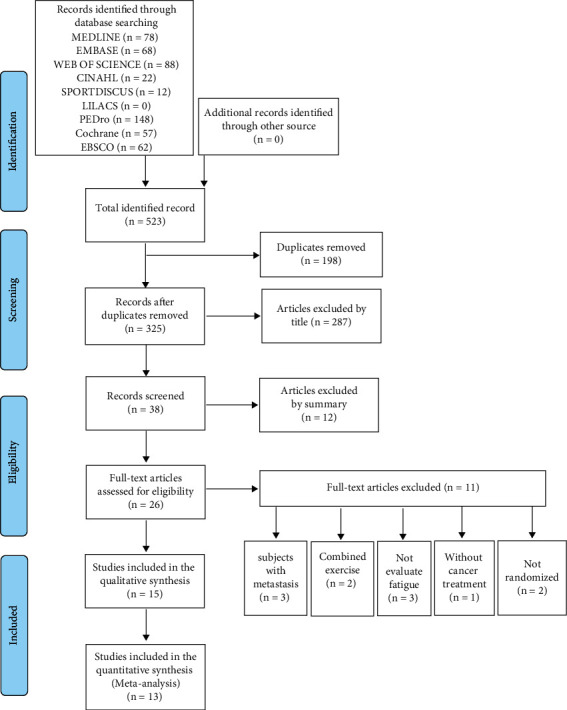
PRISMA flow diagram for the systematic review.

The electronic search identified a total of 523 studies across 8 scientific databases, and 15 randomized clinical trials were included. Details on the selection of studies are in Figure 1.

### 3.2. Study Characteristics

The characteristics of the fifteen studies selected are shown in Table 1. The sample studied in general included 1439 adults with an age range from 18 years to 78 years old. Regarding cancer type, 6 articles were found on subjects with stage 0-III breast cancer [28–33], two on subjects with head-neck cancer [34, 35], four on subjects of prostate cancer [36–39], two articles on subjects with hematological neoplasms (leukemia, lymphoma, multiple myeloma, and myelodysplastic syndrome) [40, 41], and one article on subjects of germ cell cancer [42]. According to the type of cancer treatment, in five articles, subjects were undergoing chemotherapy [28–31, 41]; in seven clinical trials, the sample received radiotherapy [28, 32, 33, 37, 38]; in two articles, the subjects were undergoing hematopoietic stem cell transplantation [40, 41]; and in two studies, the subjects underwent androgen deprivation therapy [36, 39].

Regarding the intervention group, the duration of the RT was seven [34], six [40, 41], eight [38], nine [42], twelve [28–31, 35, 36], and twenty-four weeks [37, 39]. Regarding the RT characteristics, in five studies, a RT with resistance bands [35, 38–41] was performed and nine articles performed a progressive RT [29–34,36, 37, 42]. With regard to the progressive training load, 60%-70% of 1RM [28–30, 36, 37], 60%-80% 1RM [31–33], and 15-12% RM [42] were used; Grote et al.'s study did not specify the percentage of 1RM [34]. Only the study by Cheng et al. uses traditional strength training using 60% 1RM [28] (Table 1).

The control group in 12 studies did not receive any type of intervention, and the subjects continued their daily activities and medical care as usual [28–30, 33–38, 40–42]. In two articles, the control group subjects received an intervention corresponding to progressive muscular relaxation according to Jacobson [31]. Only in one study did the control group perform aerobic training [39] (Table 1).

### 3.3. Quality Assessment

Regarding the methodological quality of the primary studies, 86.6% presented a good methodological quality and 13.3% acceptable methodological quality. According to critical criteria, the sample was randomly assigned in all the selected studies, the allocation was concealed in 66.6%, and in 33.3% of the articles, it was not concealed; the groups were similar at the baseline regarding the most important prognostic indicators in all studies. Regarding the criteria for blinding the therapist and subjects, these were not met in all the selected articles; likewise and in relation to the blinded evaluators, only two study met this criterion [36, 42]. Details of the methodological quality of the studies are presented in a supplementary file.

The effect of the intervention on fatigue levels is described in detail in Table 2. Three articles reported lower fatigue levels in the group that underwent progressive resistance training compared to a control group [36, 37, 42] and compared to the group that underwent muscle relaxation [33]. In addition, only tree studies that used a resistance training with resistance bands showed to be effective to reduce the fatigue compared to the control group [28, 38, 41]. On the other hand, nine clinical trials that used RT as an intervention did not report significant changes in fatigue levels of the intervened subjects [29–32, 34, 35, 39, 40].

### 3.4. Results of Individual Studies

Thirteen out of the fifteen selected studies presented the mean and standard deviation to calculate the ES of the intervention [28–31, 33–42]. The articles that demonstrated positive effects for fatigue levels have an ES between -0.47 and 1.17, with CI values of the studies ranging from -1.01 to 2.09 [31, 33, 36–38, 41, 42]. The effectiveness of resistance training to reduce fatigue levels based on the GRADE approach is presented in Tables 3 and 4.

### 3.5. Meta-Analysis

**Figure 2:**
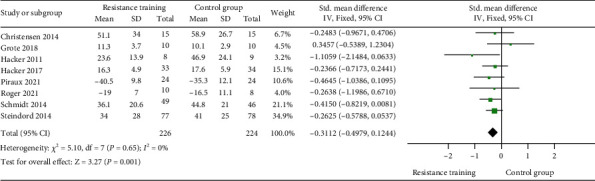
Forest plot of 10- to 35-session muscle strength exercise training versus no intervention for fatigue levels.

Eight studies reported data that allowed quantifying the effects of resistance training lasting from 10 to 35 sessions [31–35, 38, 40–42]. The pooled standardized mean difference (SMD) showed a significant reduction in fatigue levels after resistance training in people with cancer compared to the control group (SMD = −0.31; CI 95% = −0.50 and -0.12; *P* = 0.001), without significant heterogeneity (*I*^2^ = 0%; *P* = 0.65) (Figure 2).

**Figure 3:**
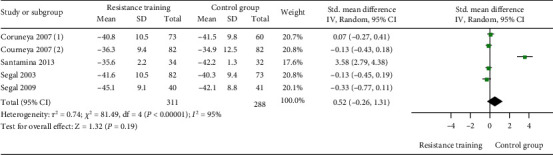
Forest plot of greater than 36-session muscle strength exercise training versus no intervention for fatigue levels.

Five studies reported data that allowed quantifying the effects of resistance training that lasted more than 35 sessions [29, 30, 36, 37, 39]. The pooled SMD estimated did not show significant changes in postexercise fatigue levels in people with cancer compared to the control group (SMD = 0.52; CI 95% = −0.26 to 1.31; *P* = 0.19), with significantly high heterogeneity (*I*^2^ = 95%; *P* ≤ 0.00001) (Figure 3).

### 3.6. Publication Bias

**Figure 4:**
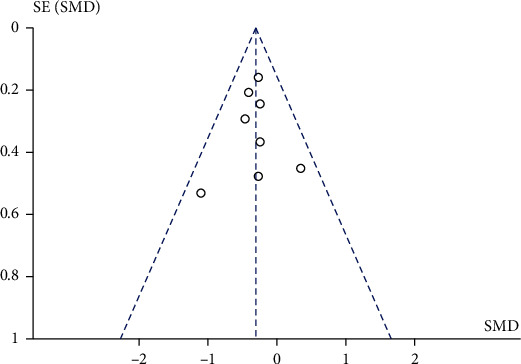
Funnel plot of eight studies that performed an intervention of 10 to 35 training sessions with muscular strength exercises.

**Figure 5:**
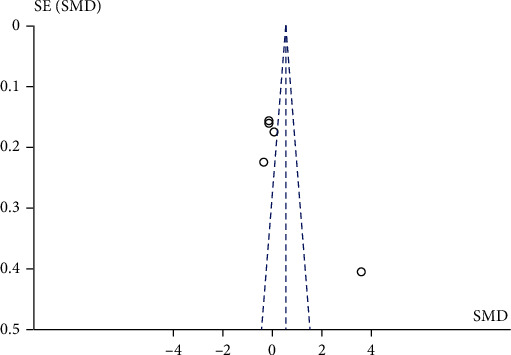
Funnel plot of five studies that performed an intervention with more than 35 training sessions with muscular strength exercises.

Publication bias was estimated for eight studies that performed an intervention of 10 to 35 sessions. According to the funnel plot, there is no evident asymmetry, therefore indicating an absence of publication bias in this meta-analysis (Figure 4). On the other hand, five studies that carried out an intervention of more than 35 sessions have a publication bias, since all the studies are the outside area in the funnel plot (Figure 5).

### 3.7. Sensitivity Analysis

A sensitivity analysis was performed to test the effect of studies with fair methodological quality and studies with smaller sample sizes. As for the influence of studies with regular methodological quality [41], the results did not change in favor of resistance training compared to the control groups and the SMD was reduced from -0.31 to -0.28, without significant heterogeneity (*I*^2^ = 0%; *P* = 0.83). Likewise, regarding studies with a small sample size [34, 35, 41], these do not influence the results reported in the meta-analysis; the SMD changes from -0.31 to -0.32 without significant heterogeneity (*I*^2^ = 0%; *P* = 0.95).

## 4. Discussion

The objective of the present systematic review and meta-analysis was to evaluate the effectiveness of resistance training to better the fatigue levels in cancer patients that are undergoing adjuvant and/or neoadjuvant treatment.

In relation to the quantitative analysis, the results of the meta-analysis show that resistance training with 10 to 35 sessions using elastic bands and/or an external load between 60% and 80% of the 1RM generates significant changes to reduce fatigue levels compared to a control group. On the other hand, the group intervened with resistance training with a number equal to or greater than 35 sessions did not generate significant changes in fatigue levels compared to the control group.

The findings reported in this systematic review are like those obtained in two published meta-analyses [17, 18]. The study by Hilfiker et al. [17] reported that any type of exercise significantly reduces fatigue levels in subjects who are receiving adjuvant/neoadjuvant therapy or after cancer treatment. Likewise, the study by Kessels et al. [18] reported the positives effects of aerobic exercises and combined exercises in reducing fatigue. Even though both meta-analyses [17, 18] support exercises to reduce fatigue, they lack an analysis regarding the specificity of resistance exercise, dosage, and the level of scientific evidence. However, despite the lack of specificity in terms of dosage, exercise and physical activity have been shown to significantly reduce fatigue related to any type of cancer [43].

The etiology of fatigue related to cancer and cancer treatment is still not completely clear; however, there are multiple causal mechanisms that have been postulated, such as emotional and cognitive factors, genetic variants, proinflammatory mechanisms, immune response, and molecular mechanisms involved in cachexia that generates neuromuscular complications and that could further exacerbate fatigue [44]. Regarding proinflammatory mechanisms, it has been hypothesized that the beneficial effects of exercise are related to the release of IL-6 myokine from the muscle, which generates negative feedback on the production of proinflammatory cytokines IL1-*β* and TNF-*α* [45]. Only one study [32] included in this systematic review reported that resistance exercise reduces proinflammatory makers associated with a reduction in physical fatigue in subjects with breast cancer. However, no specific data were reported on fatigue levels. Likewise, a pervious study [46] reported an inverse correlation between improved strength/muscle mass with reduced inflammation and decreased fatigue levels in survivors of breast cancer subjects trained with resistance exercise. Therefore, resistance exercise through its benefits in reducing proinflammatory markers could reduce fatigue levels in cancer patients. However, there is a lack of more randomized clinical trials that provide more specific and categorical estimates of mechanisms involved in reducing fatigue.

Resistance exercise can play an important role in cancer patients, since through the reduction in inflammation previously mentioned, the decrease in catabolism, and the increase in satellite cells in type II muscle fibers, it allows reducing muscle atrophy related to cachexia in cancer [47, 48]. According to the above, adequate function and muscle mass generate significant improvements in fatigue symptoms, reduce the side effects of cancer treatment, reduce depression, improves the quality of life, and increases survival in patients undergoing resistance training [49, 50].

The results of the systematic review and meta-analysis reported an update of the kinesiology clinical practice, with a synthesis at moderate to high level of evidence according to GRADE on the effectiveness of resistance training of 10 to 35 sessions to reduce fatigue levels in cancer with a moderate to strong magnitude of the effect. Likewise, the synthesis of the evidence according to GRADE is high on the effectiveness of resistance training greater than or equal to 36 sessions to reduce fatigue levels in cancer with minimal to moderate effect magnitude.

The clinical guidelines have established that resistance exercise is safe for cancer patients either during or after cancer treatment. It is recommended at a moderate intensity and a frequency of 2-3 times a week, involving large muscle groups; however, the lack of personalization of the exercise regimes is a limitation [51]. Future clinical trials should consider for targeted planning of each patient and a more specific evaluation that considers critical variables such as physical fitness, lifestyle habits such as a sedentary lifestyle and smoking, comorbidities present in the patient, cancer type, and treatment regimens as well as possible side effects of cancer treatment. In addition, they should consider the chronic effects of resistance exercise on fatigue levels, with long-term follow-up.

The limitations of this systematic review and meta-analysis are mainly related to the following: (1) different volumes of training and exercise protocols were used in the primary studies; (2) few studies greater than or equal to 36 sessions were incorporated into the meta-analysis; (3) moderate heterogeneity and publication bias of studies greater than or equal to 36 sessions may indicate variability in the estimation of the effect; (4) three included studies had a small sample size which could influence a possible overestimation; (5) two studies did not show descriptive data for the main variable; (6) different types of questionnaires were used for evaluating fatigue; (7) different cancer treatments were employed in the sample; and (8) the sample presents different types of cancer.

## 5. Conclusions

Based on the limitations of the current evidence mentioned above, we recommend for future research to conduct randomized clinical trials with larger sample size and duration of resistance training greater than 36 sessions in people with cancer during oncology treatment.

Despite the above limitations, it can conclude that resistance training of 10 to 35 sessions is effective in reducing fatigue levels in cancer subjects who are undergoing cancer treatment with a moderate quality level of evidence.

## Figures and Tables

**Table 1:** Characteristics of the studies included in the review.

Author and year	Characteristic of the population		Characteristic of the intervention			Methodological quality
	Sample size and age	Cancer type and cancer treatment	Type of intervention	Duration and frequency	Training schedule (1RM percentage, sets, repetitions, and weight increase)	PEDRo scale total score
Neumann et al., [27]	*N* = 201; 25-78 years: EG (*n* = 73), EG (*n* = 68), and CG (*n* = 60)	I–IIIa breast cancer stage Chemotherapy	EG1: progressive muscle strength training. EG2: traditional aerobic training. CG: no intervention	12 weeks; 3 times per week	EG1: 2 sets, 8-12 repetitions, 60-70% 1RM, and 10% weight increase when they complete >12 repetitions. EG2: 60% of VO_2_ max for 15 min and progressing at 80% of VO_2_ max for 45 minutes	6

Courneya et al., [29, 30]	*N* = 242; EG1 (*n* = 82; 49.5 years), EG2 (*n* = 78; 49 years), and CG (*n* = 82; 49 years)	I–IIIa breast cancer stage Chemotherapy	EG1: progressive muscle strength training. Leg extension, leg curl, leg press, calf raise, chest press, seated row, triceps extension, bicep curl, and modified abdominal. EG2: traditional aerobic training. CG: no intervention	12 weeks; 3 times per week	EG1: 2 sets, 8-12 repetitions, 60-70% 1RM, and 10% weight increase when they complete >12 repetitions. EG2: 60% of VO_2_ max for 15 min and progressing at 80% of VO_2_ max for 45 minutes	7

Cheng et al., [28]	*N* = 78; age ≥ 55 years: EG1 (*n* = 27), EG2 (*n* = 25), and CG (*n* = 26)	Lung, gastric, and breast cancer Chemotherapy, radiotherapy	EG1: traditional muscle strength training. Standing row, bench press, standing upper limbs dumbbell press, lying leg lifts, prone leg raises, and prone leg curls. EG2: traditional muscle strength training. Standing row, bench press, standing upper limbs dumbbell press, lying leg lifts, prone leg raises, and prone leg curls CG: no intervention	12 weeks; 3 times per week	EG1: 10 sets, 3 min each, and 30% 1RM; EG2: 10 sets, 3 min each, and 60% 1RM	6
Christensen et al., [42]	*N* = 30; EG1 (*n* = 15; 34.4 ± 7.6 years) and CG (*n* = 15; 35.8 ± 8.9 years)	Testicular germ cell cancer Chemotherapy	EG1: progressive muscle strength training. Leg press, knee extension, chest press, and lateral pull down using stationary equipment (Technogym) CG: no intervention	9 weeks; 3 times per week	EG1: 3 sets, 15-10 repetitions, and 15-12 RM	6

Grote et al., [34]	*N* = 20; EG (*n* = 10; 60.2 ± 4.7 years) and CG (*n* = 10; 61.5 ± 15.7 years)	Head-neck cancer stages Radiotherapy	EG: progressive muscle strength training. Leg press, chest pull, and chest press (Kaphingst). CG: no intervention	7 weeks; 3 times per week	EG: 3 sets and 8-12 repetitions. Weight load increased when the PER > 7. 2.5 kg load increased in upper extremities and 5.0 kg in lower extremities	6

Hacker et al., [41]	*N* = 19, 46.26 ± 16.23 years: EG (*n* = 9) and CG (*n* = 10)	Malignant neoplasm Hematopoietic stem cell transplantation	EG: muscle strength training with elastic bands. Chest fly, bicep curl, triceps extension, knee bend, shrug, vertical shoulder row, lateral shoulder raise, knee bend, knee extension, wall push-up, squats, and in-bed sit-ups. CG: no intervention	6 weeks; 2 times per week	EG: 1-2 sets, 8-10 repetitions, and elastic resistance band. The progression of exercise was determined by increasing the resistance of the elastic band according to the Borg scale	4

Hacker et al., [40]	*N* = 67; EG (*n* = 33; 51.9 ± 12.7 years) and CG (*n* = 34; 54.6 ± 11.6 years)	Leukemia, lymphoma, multiple myeloma myelodysplastic syndrome Hematopoietic stem cell transplantation	EG: muscle strength training with elastic bands. Seated leg press, seated row machine, trunk flexion, knee flexion machine, bench press, trunk extension machine, push press, standing planted flexion, and frontal pulldown. CG: no intervention	Hospitalized 2 times per week. 6 weeks; 3 times per week after hospital discharge	EG: 1-2 sets and elastic resistance band. The progression of exercise was determined by increasing the resistance of the elastic band according to the Borg scale	6

Piraux et al., [38]	*N* = 72; EG1 (*n* = 24; 67.9 ± 7.1 years), EG2 (*n* = 24; 67.4 ± 8.9 years), and CG (*n* = 24; 71.9 ± 8.1 years)	Prostate cancer Radiotherapy	EG1: muscle strength training with elastic bands. Abdominal, pectoral, deltoid, trapezius, latissimus dorsi, erector spinae, biceps, triceps, quads, hamstrings, gastrocnemius, soleus, and glutes. EG2: aerobic training type HIIT. CG: no intervention	5-8 weeks; 3 times per week	EG1: 3 sets, 8-12 repetitions, and body weight; elastic bands were used. EG2: 5 minutes: 65–70% HR max. 8 × 60 s ≥ 85%HR max	6

Rogers et al., [35]	*N* = 15; EG (*n* = 7; 54.7 ± 10.6 years) and CG (*n* = 8; 65.5 ± 12.5 years)	Head-neck cancer Radiotherapy	EG: muscle strength training with elastic bands. Chest press, leg extension, lateral row, reverse curl, triceps using wall push-ups or triceps kickback, heel raise, 2-arm frontal raise, hamstring curl, and arm curl. CG: no intervention	12 weeks; 2 times per week	EG: 1 set, 10 repetitions, and resistance bands. Light, moderate, and heavy resistance bands were used	7

Santa Mina et al., 2013	*N* = 66; EG (*n* = 34; 70.6 ± 9.5 years) and CG (*n* = 32; 72.1 ± 8.9 years)	Prostate cancer Androgen deprivation therapy	EG: muscle strength training with elastic bands. The resistance exercises were ball squats, hamstring curls, push-ups, upright rows, triceps extensions, bicep curls, seated row, lateral raises, abdominal crunches on the ball, and hip extensions. CG: aerobic exercise training	6 months; 3 times per week	EG: 2-3 sets and 8-12 repetitions each at an intensity of 12–15 on a rating of perceived exertion scale; elastic bands were used. CG: 30-60 min/session, 60–80% of heart rate reserve	6

Schmidt et al., [31]	*N* = 95; EG (*n* = 49; 52.7 ± 10 years) and CG (*n* = 46; 52.2 ± 9.9 years)	Breast cancer Chemotherapy	EG: progressive muscle strength training. 8 machine-based exercises; CG: progressive muscle relaxation according to Jacobson	12 weeks; 2 times per week	EG: 3 sets, 8-12 repetitions, and 60-80% 1RM	7

Schmidt et al., [32]	*N* = 103; EG (*n* = 54; 57.1 ± 8.9 years) and CG (*n* = 49; 57.3 ± 8.8 years)	0-III breast cancer stage Radiotherapy	EG: progressive muscle strength training. 8 machine-based exercises; CG: progressive muscle relaxation according to Jacobson	12 weeks; 2 times per week	EG: 3 sets, 8-12 repetitions, and 60-80% 1RM	4

Segal et al., [36]	*N* = 155; EG: (*n* = 82; 68.2 ± 7.9 years) and CG (*n* = 73; 67.7 ± 7.5 years)	Prostate cancer Androgen deprivation therapy	EG: progressive muscle strength training+placebo. Leg press, chest press, leg extension, leg curl, shoulder press, seated side pull row, calf raise, crunch, and back extension. CG: no intervention	12 weeks; 3 times per week	EG1: 2 sets, 8-12 repetitions, and 60%-70% 1RM. Increased resistance by 5 pounds when more than 12 repetitions were completed	8

Segal et al., [37]	*N* = 121; EG1: (*n* = 40; 66.4 ± 7.6 years), EG2 (*n* = 40; 66.2 ± 6.8 years), and CG (*n* = 41; 65.3 ± 7.6 years)	Prostate cancer Radiotherapy	EG1: progressive muscle strength training+placebo. Leg press, chest press, leg extension, leg curl, shoulder press, seated side pull row, calf raise, crunch, and back extension. EG2: traditional aerobic training. CG: no intervention	24 weeks; 3 times per week	EG1: 2 sets, 8-12 repetitions, and 60%-70% 1RM. Increased resistance by 5 pounds when more than 12 repetitions were completed. EG2: weeks 1 to 4: 50%-60% VO_2_ max Weeks 5 to 24: 70%-75% VO_2_ max	7

Steindorf et al., [33]	*N* = 155; EG (*n* = 77; 55.2 ± 9.5 years) and CG (*n* = 78; 56.4 ± 8.7 years)	0-III breast cancer stage Radiotherapy	EG: progressive muscle strength training. Leg extension, leg curl, leg press, internal and external shoulder rotation, seated row, downward latissimus pulls, shoulder flexion and extension, and butterfly and reverse butterfly. CG: no intervention	12 weeks; 2 times per week	EG: 3 sets, 8-12 repetitions, and 60%-80% 1RM. Increase in load by 5% in all 3 sets of 12 repetitions was successfully completed	7

EG: experimental group; CG: control group; 1RM: one-repetition maximum; HIIT: high-intensity interval training; PER: perceived exertion rating; VO_2_ max: maximum oxygen consumption; HR: heart rate.

**Table 2:** Effects of muscle strength exercise on fatigue.

Author and year	Methodology used to evaluate fatigue		Results	
	Trained group	Scale used to evaluate fatigue	Percentage variation in fatigue due to resistance training (mean ± standard deviation)	Fatigue/effect size
Courneya et al., [29, 30]	EG: traditional muscle strength training. Leg extension, leg curl, leg press, calf raise, chest press, seated row, triceps extension, bicep curl, and modified abdominal	FACT-An	EG: (pre 34.3 ± 10.1 vs.post 40.8 ± 10.5) = +18.9%	FACT (=)/intergroup ES: 0.07 (-0.28; 0.41); intragroup ES: -0.63 (-0.91; -0.25)
Courneya et al., [29, 30]	EG: traditional muscle strength training	FACT-An	EG: (pre 42.3 ± 12 vs.post 36.4 ± 12.7) = −13.9%	FACT (=)/intergroup ES: -0.13 (-0.42; 0.19); intragroup ES: -0.20 (-0.50; 0.12)
Cheng et al., [28]	EG: traditional muscle strength training. Standing row, bench press, standing upper limbs dumbbell press, lying leg lifts, prone leg raises, and prone leg curls	BFI	EG: *P* < 0.05	BFI (+)/TE: DN
Christensen at al., [42]	EG: progressive muscle strength training. Leg press, knee extension, chest press, and lateral pull down using stationary equipment (Technogym)	Fatigue subscale of EORTC-QLQ-C30	EG: (pre 26.2 ± 26.4 vs.post 51.1 ± 34.0) = +48.7%	EORTC QLQ-C30 (+)/intergroup ES: 0.26 (-0,48; 0.95); intragroup ES: -0.82 (-1.50; -0.01)
Grote et al., [34]	EG: traditional muscle strength training. Leg press, chest pull, and chest press (Kaphingst).	MFI	General fatigue EG: (pre 11.3 ± 3.7 vs.post 11.8 ± 4.3) = +4.4%; physical fatigue EG: (pre 12.0 ± 5.0 vs.post 13.3 ± 5.0) = +10.8%; mental fatigue EG: (pre 7.6 ± 4.9 vs.post 8.3 ± 2.3) = +9.2%	MFI (=)/intergroup ES: -0.36 (-1.22; 0.55); intragroup ES: -0.12 (-0.99; 0.76)
Hacker et al., [41]	EG: traditional muscle strength training. Chest fly, bicep curl, triceps extension, knee bend, shrug, vertical shoulder row, lateral shoulder raise, knee bend, knee extension, wall push-up, squats, and in-bed sit-ups	Fatigue subscale of EORTC-QLQ-C30	EG: (pre 30.6 ± 15.4 vs.post 23.6 ± 13.9) = −22.8%	EORTC QLQ-C30 (+)/intergroup ES: 1.17 (0.06; 2.09); intragroup ES: 0.48 (-0.55; 1.43)
Hacker et al., [40]	EG: traditional muscle strength training. Seated leg press, seated row machine, trunk flexion, knee flexion machine, bench press, trunk extension machine, push press, standing planted flexion, and frontal pulldown	CFQ fatigue subscale of EORTC-QLQ-C30	CFQ: general fatigue EG: (pre 16.9 ± 6.1 vs.post 16.3 ± 4.9) = −3.5%; physical fatigue EG: (pre 11.7 ± 4.3 vs.post 11.7 ± 3.9) = 0%; mental fatigue EG : (pre 5.2 ± 2.4 vs.post 4.5 ± 1.9) = −13.4%; EORTC QLQ-C30: EG: (pre 38.7 ± 28.9 vs.post 41.8 ± 24.9) = +8%	Chalder fatigue scale (=)/intergroup ES: 0.24 (-0.26; 0.70); intragroup ES: 0.11 (-0.38; 0.58); EORTC-QLQ-C30 (=)/intergroup ES: 0.07 (-0.42; 0.54); intragroup ES: -0.11 (-0.59; 0.38)
Piraux et al., [38]	EG: traditional muscle strength training. Abdominal, pectoral, deltoid, trapezius, latissimus dorsi, erector spinae, biceps, triceps, quads, hamstrings, gastrocnemius, soleus, and glutes	FACIT-F	EG: (pre 41.2 ± 7.7 vs.post 40.5 ± 9.8) = −1.6%	FACIT-F (+)/intergroup ES: -0.47 (-1.01; 0.14); intragroup ES: 0.08 (-0.49; 0.64)
Rogers et al., [35]	EG: traditional muscle strength training. Chest press, leg extension, lateral row, reverse curl, triceps using wall push-ups or triceps kickback, heel raise, 2-arm frontal raise, hamstring curl, and arm curl	FACT-F	EG: (pre 14.4 ± 6.7 vs.post 19.0 ± 10.0) = +24%	FACT-F (=)/intergroup ES: -0.24 (-1.23; 0.80); intragroup ES: -0.58 (-1.60; 0.53)
Santa Mina et al., [39]	EG: progressive muscle strength training. The resistance exercises were ball squats, hamstring curls, push-ups, upright rows, triceps extensions, bicep curls, seated row, lateral raises, abdominal crunches on the ball, and hip extensions	FACT-F	EG: (pre 38.1 ± 2.1 vs.post 35.6 ± 2.2) = −6.7	FACT-F (=): intergroup ES: 3.63 (2.6; 4.09); intragroup ES: 1.16 (0.56; 1.58)
Schmidt et al., [31]	EG: progressive resistance exercises. 8 machine-based exercises	FAQ	Total fatigue EG: (pre 36.4 ± 19.2 vs.post 36.1 ± 20.6) = −0.8%; physical fatigue EG: (pre 40.4 ± 24.5 vs.post 39.9 ± 25.0) = −1.2%; affective fatigue EG: (pre 29.2 ± 21.9 vs.post 26.8 ± 23.5) = −8.2%; cognitive fatigue EG: (pre 30.2 ± 25.3 vs.post 34.9 ± 25.1) = +13.4%	FAQ (=)/intergroup ES: 0.42 (-0.02; 0.79); intragroup ES: 0.02 (-0.38; 0.41)
Schmidt et al., [32]	EG: traditional resistance exercises. Leg extension, leg curl, seated chest curl, latissimus curl, shoulder press, triceps extension, bicep curl, calf raise, lower back extension, and modified push-ups	FAQ	EG: *P* < 0.05	FAQ (=)/TE: DN
Segal et al., [36]	EG: progressive resistance exercises. Leg press, chest press, leg extension, leg curl, shoulder press, seated side pull row, calf raise, crunch, and back extension	FACT-F	EG: (pre 40.8 ± 10.6 vs.post 41.6 ± 10.5) = +1.9%	FACT-F (+)/intergroup ES: -0.13 (-0.44; 0.20); intragroup ES: -0.08 (-0.38; 0.24)
Segal et al., [37]	EG: progressive resistance exercises. Leg press, chest press, leg extension, leg curl, shoulder press, seated side pull row, calf raise, crunch, and back extension	FACT-F	EG: (pre 42.8 ± 8.7 vs.post 45.1 ± 9.1) = +5%	FACT-F (+)/intergroup ES: -0.34 (-0.75; 0.13); intragroup ES: -0.26 (-0.68; 0.20)
Steindorf et al., [33]	EG: progressive resistance exercises. Leg extension, leg curl, leg press, internal and external shoulder rotation, seated row, downward latissimus pulls, shoulder flexion and extension, and butterfly and reverse butterfly	Fatigue subscale of EORTC-QLQ-C30 FAQ	EORTC-QLQ-C30 EG: (pre 42 ± 25 vs.post 34 ± 28) = −19%; FAQ total fatigue EG: (pre 5.9 ± 2.2 vs.post 5.4 ± 2.3) = −8.4%; physical fatigue EG: (pre 5.7 ± 2.7 vs.post 5.0 ± 2.8) = −12.2%; affective fatigue EG: (pre 5.8 ± 2.7 vs.post 5.3 ± 2.6) = −8.6%; cognitive fatigue EG: (pre 4.9 ± 3.0 vs.post 4.9 ± 3.2) = +0.2%	EORTC-QLQ-C30(=)/intergroup ES: 0.26 (-0.07; 0.56); intragroup ES: 0.30 (-0.04; 0.60); FAQ (+)/intergroup ES: 0.36 (0.01; 0.65); intragroup ES: 0.25 (-0.08; 0.55)

EG: resistance training group; HIIT: high-intensity interval training; EORTC-QLQ-C30: European Organization for Research and Treatment of Cancer Core 30-Item Quality of Life Questionnaire; PFS: Piper Fatigue Scale; FACT-An: the Functional Assessment of Cancer Therapy-Anemia; MFI: the Multidimensional Fatigue Inventory; CFQ: Chalder fatigue scale; FACT-F: Functional Assessment of Cancer Therapy Fatigue; FAQ: fatigue assessment questionnaire; DN: data not described; ES: effect size; (+): positive treatment effect; (=): treatment without effect.

**Table 3:** Synthesis of GRADE evidence of 15 to 35 sessions of a muscle strength training on fatigue levels.

Certainty evaluation							No. of patients		Absolute (CI 95%)	Certainty	Importance
No. of trials	Trial design	Risk of bias	Inconsistency	Indirect evidence	Imprecision	Risk of Publication	10 to 35 training sessions	Control group	Median 1.31 (-2.61; 5.01)	⨁⨁⨁Moderate	○8 = critical
10^a^	Random trials	Not serious	Not serious	Not serious	Serious	Serious	435/654 (66.5 %)	300/654 (45.8 %)			

^a^Cheng et al. [28]; Christensen et al. [42]; Grote et al. [34]; Hacker et al. [41]; Hacker et al. [40]; Rogers et al. [35]; Schmidt et al. [31]; Schmidt et al. [32]; Steindorf et al. [33]; Piraux et al. [38]. CI: confidence interval.

**Table 4:** Synthesis of GRADE evidence of 36 or more sessions of muscle strength training on fatigue levels.

Certainty evaluation							No. of patients		Absolute (CI 95%)	Certainty	Importance
No. of trials	Trial design	Risk of bias	Inconsistency	Indirect evidence	Imprecision	Risk of Publication	Greater than or equal to 33 training sessions	Control Group	Median 3.10 (0.71; 5.45)	⨁⨁⨁⨁High	8 = critical
5^a^	Random trials	Not serious	Not serious	Not serious	Not serious	Serious	451/785 (57.4%)	288/785 (36.6%)			

^a^Courneya et al. [29, 30]; Courneya et al. [29, 30]; Segal et al. [37]; Segal et al. [36]; Santa Mina et al. [39]. CI: confidence interval.

## Data Availability

All data are presented in Results and supported by the supplemental file.

## Supplementary Materials

Supplementary Materials Search strategy. Form for data collection of studies. Supplementary Table 1: identification of included studies in the review ranked according to the PEDro scale.
