# Supplementary material for: Effectiveness of Resistance Training on Fatigue in Patients Undergoing Cancer Treatment: A Meta-Analysis of Randomized Clinical Trials
Source: Int J Breast Cancer. 2022 Aug 8;2022:9032534. doi: 10.1155/2022/9032534 (PMC9378000; doi:10.1155/2022/9032534)
Supplement: Supplementary Materials — Search strategy. Form for data collection of studies. Supplementary Table 1: identification of included studies in the review ranked according to the PEDro scale. [file 9032534.f1.docx]

## **Medline search strategy**

1. Cancer

2. radiotherapy

3. chemotherapy

4. #2 OR #3

5. fatigue

6. "resistance exercise"

#1 AND #4 AND #5 AND #6

**EMBASE search strategy**

1. fatigue AND cancer AND (radiotherapy OR chemotherapy) AND 'resistance exercise'

#1 AND 'randomized controlled trial'/de AND 'article'/it

**WEB OF SCIENCE** **search strategy**

AB=(fatigue AND cancer AND (radiotherapy OR chemotherapy) AND 'resistance exercise')

**Cocrhane Library search strategy**

1. ((Cancer) AND (Fatigue) AND ("resistance exercise training")):ti,ab,kw

**EBSCO- sportidicus – CINHAL search strategy**

1. fatigue AND cancer AND (radiotherapy OR chemotherapy) AND 'resistance exercise'

## **Form for data collection of studies**

| 1. **Reference (author, year, journal)** |
| --- |
| 1. **Title** |
| 1. **Study design (Randomized Controlled Trial)**    1. Groups of comparison (group intervened with resistance exercise and control group): |
| 1. **Protocol**     1. Purpose of the intervention (to reduction fatigue, to increase function, strength, and /or range of upper or lower limb)    2. Intervention (type of exercise applied, modality, duration and protocol):    3. Place of care (hospital, ambulatory, home): |
| 1. **Groups of comparison**    1. Control Group (Did they receive any treatment?)    2. Additional intervention? ( ) Yes ( ) No   What?   - Session frequency: - Duration of each session: - Supervision (Physiotherapist or Nurse): - Period of follow-up: - Dropping out of the follow-up sessions: - Immediatelly after intervention: - After the follow-up: |
| 1. **Main variable analyzed** (Functional and patient-reported outcomes related to the upper extremity, questionnaires, scales, range of motion, muscle strength, kinematics and other objective variables):   **Commonly used instruments:**   \| **Conceptual Variable (Eg.: fatigue)** \| **Used instrument**  **(Eg.:** **questionnaire type)** \| **Has reliability been tested in the use of instrumentation (between evaluators or instrument?)** \| \| --- \| --- \| --- \| \|  \|  \|  \| \|  \|  \|  \|   **Statistical test. What?**  (Main analysis to be found: Comparison between control and surgery group after treatment)  **( ) Comparisons between groups (pre to post intervention)**  **( ) Comparisons within groups (pre to post intervention)** |
| **Analysis of Difference Scores**  **Outcomes (to describe mean, standard deviation, P value and T test):**  **Clinical relevance / Magnitude of the intervention effect:** |

**Supplementary Table 1**. Identification of included studies in the review and ranked according to the PEDro scale.

| **Autor** | **1** | **2** | **3** | **4** | **5** | **6** | **7** | **8** | **9** | **10** | **11** | **Total** |
| --- | --- | --- | --- | --- | --- | --- | --- | --- | --- | --- | --- | --- |
| Courneya et al., 2007 | 1 | 1 | 1 | 1 | - | - | - | - | 1 | 1 | 1 | 6 |
| Courneya et al., 2007 | 1 | 1 | 1 | 1 | - | - | - | 1 | 1 | 1 | 1 | 7 |
| Cheng et al., 2021 | 1 | 1 | - | 1 | - | - | - | 1 | 1 | 1 | 1 | 6 |
| Christensen et al., 2014 | - | 1 | - | 1 | - | - | 1 | 1 | - | 1 | 1 | 6 |
| Grote et al., 2018 | 1 | 1 | 1 | 1 | - | - | - | 1 | - | 1 | 1 | 6 |
| Hacker et al., 2011 | 1 | 1 | - | 1 | - | - | - | - | - | 1 | 1 | 4 |
| Hacker et al., 2017 | 1 | 1 | 1 | 1 | - | - | - | 1 | - | 1 | 1 | 6 |
| Piraux et al., 2021 | - | 1 | - | 1 | - | - | - | 1 | 1 | 1 | 1 | 6 |
| Rogers et al., 2013 | 1 | 1 | 1 | 1 | - | - | - | 1 | 1 | 1 | 1 | 7 |
| Santa Mina et al., 2013 | 1 | 1 | 1 | 1 | - | - | - | - | 1 | 1 | 1 | 6 |
| Schmidt et al., 2014 | 1 | 1 | 1 | 1 | - | - | - | 1 | 1 | 1 | 1 | 7 |
| Schmidt et al., 2016 | - | 1 | - | 1 | - | - | - | - | 1 | 1 | 1 | 4 |
| Segal et al., 2003 | 1 | 1 | 1 | 1 | - | - | 1 | 1 | 1 | 1 | 1 | 8 |
| Segal et al., 2008 | 1 | 1 | 1 | 1 | - | - | - | 1 | 1 | 1 | 1 | 7 |
| Steindorf et al., 2014 | 1 | 1 | 1 | 1 | - | - | - | 1 | 1 | 1 | 1 | 7 |

1. Were the eligibility criteria specified? 2. Were the participants randomly allocated between the groups? 3. Was the allocation blinded? 4. Were the groups similar at the baseline for the most important prognostic indicators? 5. Were the participants blinded? 6. Were the therapists who performed the intervention blinded? 7. Were the evaluators who measured at least one measure of response blinded? 8. Did the measures of at least one outcome affect > 85% of the participants initially allocated to the groups? 9. Did all the participants receive the treatment or a control condition; if not, were the data analyzed with intention-to-treat analysis? 10. Did the statistical comparison results between groups report at least one key response variable? 11. Did the study present reliability measures for at least one variable response?
